# Industry-Based Misconceptions Regarding Cross-Pollination of *Cannabis* spp.

**DOI:** 10.3389/fpls.2022.793264

**Published:** 2022-01-26

**Authors:** Kenneth J. Olejar, Sang-Hyuck Park

**Affiliations:** ^1^Department of Chemistry, Colorado State University Pueblo, Pueblo, CO, United States; ^2^Institute of Cannabis Research, Colorado State University Pueblo, Pueblo, CO, United States

**Keywords:** cannabis, cross pollination, cannabinoids, pollen grain, hemp, tetrahydrocannabinol, cannabidiol

## Abstract

Cross-pollination of commercial crops has been an ongoing issue in many species. *Cannabis* spp. encompasses the classifications of marijuana [high in Δ^9^-tetrahydrocannabinol (THC)] and hemp (below 0.3% THC). As such, cannabis is the most recent crop facing the dilemma of cross-pollination and is leading to litigation. These litigations are driven by the large misunderstanding of the impacts of cross-pollination within the cannabis industry. The misconception is that if hemp is cross-pollinated by high THC cannabis, the hemp will become “hot” (high in THC) thereby rendering the crop illegal under the 2018 Farm Bill. However, there are many factors that contribute to the amount of THC a plant may produce. This article examines and refutes the misconception of cross-pollination increasing THC levels by highlighting several methods of how THC may become high in a given hemp crop.

## Introduction

The passage of the 2018 Farm Bill renewed interest in *Cannabis* spp. as a commercial crop. This interest is driven by the 565 secondary metabolites including 120 cannabinoids produced by cannabis (ElSohly et al., 2017) and their therapeutic potentials. Currently, in the United States, 24 states have approved hemp production plans with two plans under review and an additional 20 operating under the 2014 pilot program, three being USDA hemp producers (USDA Agricultural Marketing Service, 2021a). In 2020, 28,255 ha of hemp was planted in the United States, which was a decrease of 48% over the total planted hectares in 2019 (USDA Farm Service Agency, 2021).

In 2021, the USDA final rule established a domestic hemp production program in the United States and upholds the 2018 Farm Bill hemp production limits on Δ^9^-tetrahydrocannabinol (THC) concentration of less than 0.3%, which must be tested within 30 days prior to the anticipated harvest (USDA Agricultural Marketing Service, 2021b). If the %THC exceeds 0.3, the plant materials must be disposed of, costing the grower the loss of the economic value of the investment made in the crops production. As a result, litigation has become common between farmers of hemp and marijuana.

But how do these crops result in excess THC production when feminized and certified seeds are used? A main misconception within the industry is that it is caused by cross-pollination or how the crop was cultivated (Toth et al., 2020). This pollen misconception arises from *Cannabis* spp. being an annual dioecious flowering plants that produce male and female flowers on separate individuals. It is assumed that if only female plants or feminized seeds are used then the source of the problem exists external to the farm and can only be the result of cross-pollination from an illicit or legal marijuana operation. However, there are several instances that preclude this from being the cause, one being that cannabis can also be monoecious under stressful condition, thereby self-pollinating. In light of this, we will focus on several mechanisms that can account for increased THC levels in hemp crops in order to clarify the cross-pollination misconception.

## Cross-Pollination

**Figure 1:**
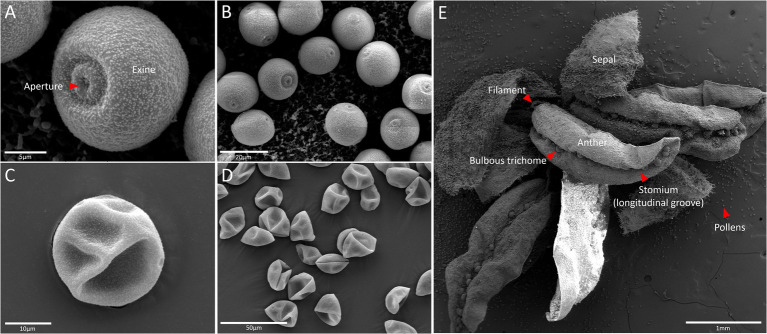
Scanning electron microscopy of fresh pollen grains **(A,B)** and dried pollens **(C,D)** of a hemp (*Cannabis sativa* L.) male flower **(E)** that comprises anther embedded with pollen sac, filament, bulbous trichome, and sepal.

Before delving into other factors that impact hemp THC levels, it is necessary to understand pollination of the cannabis plant. The anther of the male flower is responsible for the pollen in question. In theory, only one pollen grain is required to fertilize the pistil of a female plant flower, as such more than one grain significantly increases the likelihood of fertilization (Mulugeta et al., 1994). On average, the male plant produces 3,50,000 pale yellow pollen grains, thereby dramatically increasing the chances of fertilization (Figure 1). Adding to the generation of copious amounts of pollen is that the plants are exclusively wind pollinated; therefore, the pollen has evolved for maximum dispersal on the wind. As such, the risk of pollination is significant and several studies have sought to establish “safe” buffer zones between hemp crops.

Small and Antle (2003) studying a small plot, 0.4 ha, found that by 100 m pollen density had fallen to less than 1% of that found within the field (Small and Antle, 2003). They also established that the downwind dispersion was much greater than the upwind vector, thereby showing that an understanding of the wind dynamics is necessary for establishing buffer zones. A larger crop area (100–150 ha) study was conducted by Demkin and Astachova (1952), which demonstrated that the pollen density at 10 km was still 38% of the field density (Demkin and Astachova, 1952). Demkin and Astachova’s study not only demonstrates that the impact of the environmental conditions (wind) has on pollen dispersion, but also the size of the cultivated land (Demkin and Astachova, 1952). Several studies have also found cannabis pollen that has traveled from North Africa to southwestern Europe a journey of several 1,000 kms (Cabezudo et al., 1997). Holding to the notion of one pollen grain being needed to fertilize a flower, the buffer zone would preclude more than a few cultivations globally to prevent cross-pollination. However, in an attempt to be reasonable, many governments have adopted a 5 km buffer zone between hemp and marijuana cultivations (Small and Antle, 2003).

Even with the buffer zones, pollination can occur and due to the potential economic loss litigation almost certainly follows. An example is a recent litigation in Oregon where a hemp farm had to destroy its crop of female plants because of high THC levels due to cross-pollination from a neighboring marijuana cultivation containing male plants (McGuire, 2020). However, this pollination event is unlikely to be the source of the high THC levels in the growing hemp plants, while Paris et al. (1975) did find Δ^9^-tetrahydrocannabinolic acid (THCA) in pollen grains of marijuana it only amounted to 0.16 g% in pollen, well below the limit of 0.3% THC in hemp (Paris et al., 1975). Furthermore, pollination is a process by which plants increase the genetic diversity within the species. The impacts of the exchange of genetic materials are not seen in the parent plant, but in the next generation resulting from the seed of the parent plant. Consequently, a cultivation of hemp that has exceeded the legal limits of THC is only the result of cross-pollination when the seed used is the result of cross-pollination.

To avoid the potential of seeds with questionable genetics, many farmers have turned to clones. This practice also has the added benefit of ensuring that the cultivation is of female plants. The clones all carry the desired traits of the originally selected “mother” plant and are genetically identical in morphological and chemical characteristics. However, in outdoor grows, these clones do become pollinated and can exceed the allowable THC limits. This would seem to support the antidotal suggestion that cross-pollination is the cause; however, other factors are at play. Abiotic and biotic stressors are the most likely cause of these high THC instances (Hakim et al., 1986) and will be examined later in the article.

But how can we be certain that pollination does not account for the THC levels? Examining the metabolic properties of plants once pollinated it is found that there is a shift in the secondary metabolites (Flores-Sanchez and Verpoorte, 2008). However, these shifts are not toward the production of one or two specific compounds, but an across the board shift to lower secondary metabolite production as a result of the shift in energy to reproduction. Hence, while a cannabis plant typically produces secondary metabolites in response to abiotic and biotic stressors, once pollination has occurred the focus of energy is shifted to seed production not cannabinoid production.

## Abiotic and Biotic Stressors

Cannabinoids are secondary metabolites, which production can be affected by various abiotic and biotic stressors with the two main cannabinoids produced being Δ^9^-THCA and cannabidiolic acid (CBDA; Andre et al., 2016; Hurgobin et al., 2021). The cannabinoids THCA and CBDA are generated *via* a common pathway utilizing cannabigerolic acid (CBGA), which is acted upon by THCA synthase or CBDA synthase to form THCA and CBDA, respectively (Tahir et al., 2021). The most common stressors impacting cannabinoid production are light, nutrition, predation, temperature, and water deficit (Desaulniers Brousseau et al., 2021). While the studies highlighted demonstrate the impacts of the specific stressor on cannabinoid production, some also delve deeper into the genetic responses that regulate the cannabinoid production demonstrating that genetics impact the final cannabinoid profile much more than the abiotic and biotic stressors.

Examining the impacts of UV-B radiation on cannabis, Lydon et al. (1987) found that floral and leaf THC levels increased following 40 days of daily exposure and no other cannabinoids in the drug-type plants (Lydon et al., 1987). The increased levels of THC following irradiation are thought to account for the physiological and morphological tolerances to UV-B radiation in drug-type plants. Similarly, Giupponi et al. (2020) found that industrial hemp grown in a mountainous region where UV light exposure was increased affected the production of CBDA and cannaflavins (Giupponi et al., 2020). Additionally, Magagnini et al. (2018) altered the light spectrum in the greenhouse and found that these variations impacted the cannabinoid content, but not the total yield (Magagnini et al., 2018). The study found that UV-A radiation increased the levels of THC (Magagnini et al., 2018). Consequently, from these studies, it can be seen that light quality can impact the levels of THC and other cannabinoids produced.

A recent study by Caplan et al. (2019) demonstrated that subjecting cannabis to drought for 11 days during flowering resulted in an increase in THC and cannabidiol (CBD) levels by 50 and 67%, respectively (Caplan et al., 2019). Expanding upon these studies, Campbell et al. (2019) examined the influence of water stress and genotype (Campbell et al., 2019). In their study, it was observed that while the environmental conditions accounted for 1.7% of the variation in THC and 6% of the variation observed in CBD. In contrast, 80% of the THC variation and 83% of the CBD variation were attributed to genetics. While the CBD and THC exhibited similar genetic control, cannabichromene (CBC) variation was explained by 50% genetics and 17% environmental (Campbell et al., 2019). The impact of environmental conditions, specifically nitrogen nutrition, water stress, and salinity, was examined in a review by Landi et al. (2019) where it is reported that multiple genes are up regulated and down regulated by these abiotic stressors thereby allowing for altered secondary metabolite production (Landi et al., 2019).

While these studies clearly demonstrate a link between environmental factors and increased cannabinoid production, with increased THC being the main concern, they also demonstrate the THC production is rooted in the genetics of the plant. The abiotic and biotic stressors are merely the triggers for up- and down regulation of the plants genes. Ultimately, to ensure that plant performs as reported, they must be grown under identical condition to how they were produced, which is nearly impossible with the varied climates where outdoor cultivation occurs. This is additionally difficult when the cannabis plants microbiome is added to the equation. Recent studies show that the microbiome can directly influence the cannabinoid production (Taghinasab and Jabaji, 2020). As such, even indoor cultivations may find difficulty in producing equivalent cannabinoid profiles to the seed manufacturer.

## Limits of Genetically Unstable Seeds

A case has been reported from a hemp grower at Yuma, Colorado that hemp plants had spiked their THC level by 3.17%, as a result the farmer had to destroy all the hemp in the field (Deventer, 2018). In most major crops, pure inbred lines are extensively used to guarantee high yields of agronomically important traits. In hemp, there are numerous hemp varieties designed for high CBD production while maintaining less than 0.3% THC. However, the CBD yields from field grown hemp plants turned out to be different than the yields claimed in the sale’s catalog because of the genetic heterozygosity of the seeds. The heterozygosity is a double-edged sword for hemp growers. In a bright side, it serves as genetic resource to develop a new variety with economically desirable traits. For example, heterozygosity is typically achieved by outcrossing from genetically unlinked two parents. Hybrid vigor or heterosis appears in the first generation of the hybrid offspring, especially in their statue, biomass, and fertility that outperform the traits of the two parents (Birchler et al., 2006). However, the heterozygosity can be problematic for hemp growers who need stable genetics for consistent high CBD production because any undesirable traits can be expressed during breeding process (Salami et al., 2020). In general, a multiple inbreeding process (>5 generations) is required to obtain a pure inbred line. To our knowledge, a few hemp varieties have reached the extent of being genetically homozygous, which means there are near zero % THC attributions in their genetics. Recent complete genome sequencing on hemp and marijuana provided the chromosomal location of THCA synthase gene that is a key enzyme to catalyze the formation of THCA from CBGA, as well as the evolutionary events of THCA genes causing copy number variation (Grassa et al., 2018, 2021; Laverty et al., 2019; Gao et al., 2020; McKernan et al., 2020). Despite gaining further insights on Cannabis genomics, the genetic regulation underlying THC biosynthesis is not fully understood because THC concentration is a polygenic trait that is determined by multiple genetic factors (Campbell et al., 2020). Most commercially available hemp seeds are heterozygous. The THC levels concentrations can readily go above 0.3% upon receiving external and internal cues, even though an initial test certifies the variety produces less than 0.3% THC. This is the most frequently observed case for growers who purchased the seeds from uncertified brokers.

Epigenetics can play a role in the modification of the genetics influencing cannabinoid production. Generally, epigenetics is characterized by histone modifications and DNA methylation, which can alter the expression of stress-responsive genes (Luo and He, 2020). This variation is thought to play an important role in the ability of plants to cope within the environment. Epigenetic expression of genes can result in up- or down regulation as stress is applied or removed and where the modification occurs in the genome (Shi et al., 2019; Miryeganeh, 2021). Epigenetic changes can result in modifications that contribute to stress memory, which can be passed into offspring (Miryeganeh, 2021). The epigenetic variation among plants can result in phenotypic variance throughout the population, contribute to plant plasticity and its ability to thrive within their environment. Consequently, this is an area of research that needs more research.

## Other Concerns (Self-Pollination and Feminized Seeds)

*Cannabis sativa* L. is a primarily dioecious plant, producing a male and female flower in a different plant. However, monoecious- or hermaphrodite plants are readily observed when cannabis is under stress. The proximity of pollen donors around female flowers is not ideal for cannabinoid production because of high probability of self-pollination which induces the energy relocation toward seed formation, resulted in lower cannabinoid yields (Thomas and ElSohly, 2016).

In this regard, female plants have been industrially exploited due to the capability of producing high cannabinoids and terpenes. Currently, numerous feminized seeds became available but the quality of seeds is in question. For example, a farmer in Montana, cultivating hemp on approximately 14,000 acres, purchased feminized seeds for a high CBD yielding hemp strain from a certified broker. Soon after they were planted, the team noticed a serious quality issue regarding the seeds. More than 30% of the planted seeds turned out to be males whose growth were inferior and cannabinoid yield were poor (*personal communication*). The failure of seed quality validation at the early stage will result in the tremendous economic loss due to the additional costs of purchasing new seeds, hiring labor, and spending time to eradicate the unwanted plants. Most importantly, the males produced pollen in that field, which introduced unwanted cross-pollination with a plant carrying an undesirable and illegal trait, THCA production.

## Conclusion

As demand for hemp production increases, several misconceptions exist among hemp growers. One of the misconceptions is that “cross-pollination” of marijuana pollens on hemp females spikes the THC concentration in hemp fields. This misconception often leads to a litigation toward neighboring marijuana growers. The altered genetics will only begin to appear in the seeds resulting from the cross-pollination, not in the pollinated female hemp plant. If seeing a “hot” hemp in the field, it might have been caused by following reasons including (1) various environmental stressors (e.g., light spectrums and water deficit), (2) using highly heterozygous seeds containing high THC allele(s), and (3) using seeds resulted from hemp that is cross-pollinated with marijuana. To comply with USDA hemp production guideline, high-quality seed selection from certified sources will be critical to ensure successful hemp/cannabinoid production. Also, close monitoring of growth condition will ensure the high yield of cannabinoids while maintaining low-THC level by minimizing the introduction of any environmental stressors. Lastly, if possible, setting up a buffer zone, even further distant than suggested, can be definitely helpful to lower the chance of cross-pollination between hemp and marijuana.

## Data Availability Statement

The original contributions presented in the study are included in the article, further inquiries can be directed to the corresponding author.

## Author Contributions

S-HP contributed to conception and wrote the first draft of the manuscript. KO reviewed and edited the manuscript. All authors contributed to manuscript revision, read, and approved the submitted version.

## Funding

This research was supported by the Institute of Cannabis Research at Colorado State University Pueblo.

## Conflict of Interest

The authors declare that the research was conducted in the absence of any commercial or financial relationships that could be construed as a potential conflict of interest.

## Publisher’s Note

All claims expressed in this article are solely those of the authors and do not necessarily represent those of their affiliated organizations, or those of the publisher, the editors and the reviewers. Any product that may be evaluated in this article, or claim that may be made by its manufacturer, is not guaranteed or endorsed by the publisher.
